# Transcriptome Profile in Dairy Cows Resistant or Sensitive to Milk Fat Depression

**DOI:** 10.3390/ani13071199

**Published:** 2023-03-29

**Authors:** Adriana Siurana, Angela Cánovas, Joaquim Casellas, Sergio Calsamiglia

**Affiliations:** 1 Animal Nutrition and Welfare Service, Autonomous University of Barcelona, 08193 Barcelona, Spain; 2 Grup de Millora Genètica Molecular Veterinària, Departament de Ciència Animal i dels Aliments, Universitat Autònoma de Barcelona, 08193 Barcelona, Spain; 3 Centre for Genetic Improvement of Livestock, Department of Animal Biosciences, University of Guelph, Guelph, ON N1G2W1, Canada

**Keywords:** milk fat depression, RNA-sequencing, single nucleotide polymorphisms

## Abstract

**Simple Summary:**

In the last 20 years, there has been interest in modifying this fatty acid profile to increase the content in polyunsaturated fatty acids which are healthier for human health. However, long chain polyunsaturated fatty acids cause milk fat depression. We observed a wide variation in the sensitivity of cows to induced milk fat depression caused by feeding on linseed-rich diets. We identified 15 genes as key gene regulators harboring SNP in cows fed the linseed-rich diet. The selected genes are novel candidates to be involved in the resistance or sensitivity of dairy cows to milk fat depression, and open the opportunity to select cows genetically resistant to milk fat depression.

**Abstract:**

Feeding linseed to dairy cows results in milk fat depression (MFD), but there is a wide range of sensitivity among cows. The objectives of this study were to identify target genes containing SNP that may play a key role in the regulation of milk fat synthesis in cows resistant or sensitive to MFD. Four cows were selected from a dairy farm after a switch from a control diet to a linseed-rich diet; two were resistant to MFD with a high milk fat content in the control (4.06%) and linseed-rich (3.90%) diets; and two were sensitive to MFD with the milk fat content decreasing after the change from the control (3.87%) to linseed-rich (2.52%) diets. Transcriptome and SNP discovery analyses were performed using RNA-sequencing technology. There was a large number of differentially expressed genes in the control (*n* = 1316) and linseed-rich (*n* = 1888) diets. Of these, 15 genes were detected as key gene regulators and harboring SNP in the linseed-rich diet. The selected genes MTOR, PDPK1, EREG, NOTCH1, ZNF217 and TGFB3 may form a network with a principal axis PI3K/Akt/MTOR/SREBP1 involved in milk fat synthesis and in the response to diets that induced MFD. These 15 genes are novel candidate genes to be involved in the resistance or sensitivity of dairy cows to milk fat depression.

## 1. Introduction

The production of omega-3-enriched milk is generally achieved by feeding oilseeds rich in these fatty acids (FA), namely linseed. Nevertheless, this production has two main problems: (a) linseed is an expensive ingredient and (b) feeding high polyunsaturated FA (PUFA) usually results in milk fat depression (MFD), which may have important economic implications due to milk price penalties. Diet-induced MFD has been typically associated to diets high in concentrate and low in fiber, or diets with high PUFA oils [1]. Although these two types of diet-induced MFD may occur independently, they can also occur simultaneously when high production dairy farms feeding diets rich in concentrate introduce PUFA to produce omega-3-enriched milk. The inhibition of milk fat synthesis resulting from ruminal biohydrogenation of FA such as trans-10, cis-12 conjugated linoleic acid in diets that induce MFD [2] has been related to the subsequent inhibition of several genes involved in de novo FA synthesis and triglyceride synthesis [3]. Moreover, some gene regulators may play an important role in milk fat synthesis [4,5,6]. We observed that in cows fed linseed on a commercial dairy farm, the average milk fat content of individual cows was highly variable. Therefore, we hypothesized that certain cows could be resistant to MFD when fed a diet supplemented with linseed. The aims of this study were (a) to compare the gene expression in milk somatic cells from cows resistant or sensitive to MFD, (b) to identify metabolic pathways and transcription factors affected by MFD in resistant or sensitive cows under different dietary conditions (i.e., no fat supplemented or linseed rich-diet) and (c) to identify target genes containing single nucleotide polymorphisms (SNP) that may play a key role in the regulation of MFD in cows resistant or sensitive to MFD.

## 2. Materials and Methods

### 2.1. Animals and Diets

This experiment was conducted in a commercial dairy farm from Catalonia (Spain) with 800 Holstein cows. Cows were on average 194 ± 12 days in milk, 34.3 ± 2.36 kg/d of milk, 3.44 ± 0.16% milk fat content and 3.35 ± 0.07% milk protein content. Before January 2013, the cows were fed a diet containing extruded linseed (6.1%; LIN), triticale silage (20.4%), brewers grains (8.6%), corn silage (10.2%), rapeseed meal (12.9%), corn grain (32.1%), soybean meal (3.0%), barley grain (2.6%) and a vitamin and mineral mixture (3.9%). Cows were then switched to a control diet with no linseed after January 2013 (CTR) and samples were taken 2 months after the change. The control diet contained triticale silage (20.4%), brewers grains (8.6%), corn silage (10.2%), rapeseed meal (12.9%), fat (1.7%), corn grain (33.8%), soybean meal (6.0%), barley grain (2.6%) and a vitamin and mineral mixture (3.9%). The diets contained similar amounts of energy (1.75 Mcal Nel/kg), 16.5% crude protein, 29.7% neutral detergent fiber, 40.0% non-fibrous carbohydrates, 5.3% fat and 8.2% ash. From four months prior to four months after the diet change, the milk samples from all cows were collected monthly and submitted to the official milk control laboratory (ALLIC, Cabrils, Spain) for milk fat, protein, lactose and somatic cell analyses by near-infrared spectroscopy. Two cows with a high milk fat content during the LIN (3.90%) and CTR (4.06%) diets (i.e., the cows resistant to milk fat depression; R-MFD), and two cows with a high fat content when fed the CTR diet (3.87%) and low fat content when switched to the LIN diet (2.52%; i.e., cows sensitive to milk fat depression; S-MFD) during at least four consecutive monthly controls were selected. The fresh milk samples of the selected cows under the LIN and CTR diets were collected for the extraction of mRNA from milk somatic cells. Medrano et al. [7] and Cánovas et al. [8] showed that milk somatic cells are representative of the mammary gland transcriptome and can be used as an alternative to tissue biopsy, a technique that is invasive and harmful to animal welfare. The mRNA extraction and processing were conducted within 4 h after sampling.

### 2.2. Sampling and Analysis

The milk samples were collected and processed as described by Wickramasinghe et al. [9]. For each cow, the milk samples were obtained by hand milking two quarters of the udder (50 mL from each quarter) before the morning milking. The samples were stored in ice and transported to the laboratory within four hours to carry out RNA extraction from milk somatic cells. Additionally, a representative 50 mL milk sample of the morning milking was also collected from each cow to analyze the FA profile by gas chromatography (ALLIC).

RNA was extracted as described by [9]. Milk somatic cells were pelleted by adding 50 µL of 0.5 M EDTA to the 50 mL fresh milk, and centrifuged at 1800 rpm at 4 °C for 10 min. The pellet was washed with 10 mL of phosphate-buffered saline at pH 7.2 and 0.5 mM EDTA and filtered through a sterile cheesecloth to remove any debris. The milk cells were centrifuged again at 1800 rpm, 4 °C for 10 min. The supernatant was decanted and RNA was extracted using the Trizol method (Invitrogen, Carlsbad, CA, USA). Purified total RNA was treated with Turbo DNase (Invitrogen, Carlsbad, CA, USA). The quality of the total RNA was evaluated using the RNA integrity number in the Agilent 2100 Bioanalyzer (Agilent Technologies, Santa Clara, CA, USA), which ranged from 6.6 to 8.8.

The mRNA was purified, fragmented and converted to cDNA as described by Cánovas et al. [10,11]. Adapters were ligated to the ends of double-stranded cDNA and PCR-amplified to create libraries. These procedures were executed with a TruSeq RNA Sample Preparation kit (Illumina, Inc., San Diego, CA, USA).

Sequencing was conducted by the Illumina HiSeq2000 (Illumina, Inc., San Diego, CA, USA) which yielded 100 bp paired sequences. The quality of obtained reads was checked with the FastQC software (http://www.bioinformatics.bbsrc.ac.uk/projects/fastqc/; accessed on 20 November 2014), as described [8]. The raw reads that passed the quality filter threshold were mapped using Tophat 2.0.7 [12] and Bowtie2 2.0.6 [13] to identify known and novel splice junctions and to generate read alignments for each sample. The Bos taurus genome 4.6.1. was used as the reference genome, and genomic annotations were obtained from Illumina’s database (http://support.illumina.com/sequencing/sequencing_software/igenome.ilmn; accessed on 30 November 2014) in general feature format 3. The inner distance between mate pairs used was 50 bp and the rest of the parameters were used with the default values. The transcript isoform level and gene level counts were calculated and reads per kilobase per million mapped reads (RPKM) were normalized using Cufflinks 2.0.2. [14]. Differential transcript expression was analyzed by the Cuffdiff program included in Cufflinks 2.0.2. The *p*-values were adjusted by the Benjamini and Hochberg method to obtain a strict control of False Discovery Rate [15]. The differential expression analysis was evaluated across the different levels of the interaction between cow type (i.e., R-MFD and S-MFD) and diet (i.e., CTR and LIN). The differentially expressed genes obtained passed a filter to eliminate the low statistical power cases [16]. In this study, the genes with an RPKM ≥ 0.2, fold change > 2 and <−2 and a *p*-value < 0.01 were selected. Ingenuity Pathway Analysis (Qiagen, Venlo, The Netherlands) was used to conduct a functional analysis to identify the metabolic pathways and key gene regulators involved in FA synthesis and lipid metabolism that explain the observed phenotype in the four comparisons conducted [17,18].

An SNP detection analysis was performed using sequencing reads from the four cows to determine putative polymorphisms in genes involved in the FA and lipid metabolism. The SNP detection was performed [10,19], considering the following quality and significance filters: (1) the minimum average quality of surrounding bases and minimum quality of the central base were set as 15 and 20 quality score units, respectively; (2) the minimum coverage was set at ten reads; (3) the minimum variant frequency or count was set at 20% or two read counts per SNP; and (4) the SNP located in read ends (last three bases) were not considered in the analysis due to possible sequencing errors.

## 3. Results and Discussion

The strength of the design relies on the fact that the analysis of differential gene expression due to the diet change were conducted in the same animals for the CTR and LIN diets, where each cow was its own control, correcting for the possibility to generate a bias. Other studies about the biology of milk traits by the RNA-sequencing differential expression also used two replicates [20]. Moreover, our sample size falls within the range of previous studies based on the RNA-sequencing techniques [21]. Given the high technical reproducibility and orders of magnitude greater resolution than gene expression microarrays, smaller sample sizes can be anticipated. Moreover, to estimate the statistical power and satisfactory sample size for RNA-sequencing differential expression, it is very challenging because of analytical complexity [22,23,24] and multiple hypotheses being tested [25]. Nevertheless, fold change has been revealed as the key factor [26] and most of the available methods failed due to small fold changes differences, not sample size. Rapaport et al. [27] also demonstrated that with most methods, over 90% of differentially expressed genes at the top expression levels could be detected with as little as two replicates and 5% of the reads. Nevertheless, a statistical power of 70% or higher can be expected under our sample size for genes with large fold change (>2 and <−2), which is the main target of our research.

### 3.1. RNA-Sequencing Expression Analysis, Pathway Analysis and Identification of Key Gene Regulators

An average of 72 million sequence reads were obtained for each sample. About 80 to 85% of them were mapped to the bovine reference sequence, and ~90% of annotated Bos taurus genes were detected (24,881 genes out of 27,368). Differential expression analyses between R-MFD and S-MFD cows detected 1316 and 1888 differentially expressed genes in CTR and LIN diets, respectively. When focusing on the differences between CTR and LIN diets, 816 and 43 genes were differentially expressed in R-MFD cows and S-MFD cows, respectively. All 43 genes reported in S-MFD cows were over-expressed when fed the LIN diet.

Differentially expressed genes were linked to 13 to 117 metabolic pathways, and 27 to 294 key gene regulators were identified. These genes were linked to the immune and inflammatory system, development and growth processes, and lipid metabolism and FA synthesis. These results suggested that S-MFD cows had a different genetic response against a linseed-rich diet compared with R-MFD cows.

### 3.2. Identification of Genes Harboring SNP

Between 25,000 and 34,000 polymorphic SNPs were detected, depending on the individual cow analysis. Of these, 6700–7300 polymorphic SNPs were identified in R-MFD cows associated with the differentially expressed genes and key gene regulator lists in the three comparisons involving R-MFD cows. In S-MFD cows, 6900–8900 polymorphic SNPs were identified associated with differentially expressed genes and key gene regulators lists in the three comparisons involving S-MFD cows. Among the polymorphic SNPs identified associated with the list of differentially expressed genes and key genes regulators, 641 polymorphic SNPs were identified only in R-MFD cows and 1024 only in S-MFD cows.

The polymorphic SNPs identified in the two selected cows and not in the other two cows, for R-MFD or S-MFD cows, were classified according to the type of function. In R-MFD cows, 63% of the polymorphic SNPs were intron-variant, 12% were synonymous codon, 7% were utr-variant-3-prime and the remaining 15% were downstream-variant-500B, missense, nc-transcript-variant, upstream-variant-2KB or utr-variant-5-prime. In S-MFD cows, 65% were intron-variant, 13% synonymous codon, 9% utr-variant-3-prime and the remaining 14% were missense, nc-transcript-variant, upstream-variant-2KB, utr-variant-5-prime, downstream-variant-500B, splice-acceptor-variant or stop-gained.

### 3.3. Identification of Target Genes Differentially Expressed, Key Genes Regulators and Contained SNP

**Figure 1:**
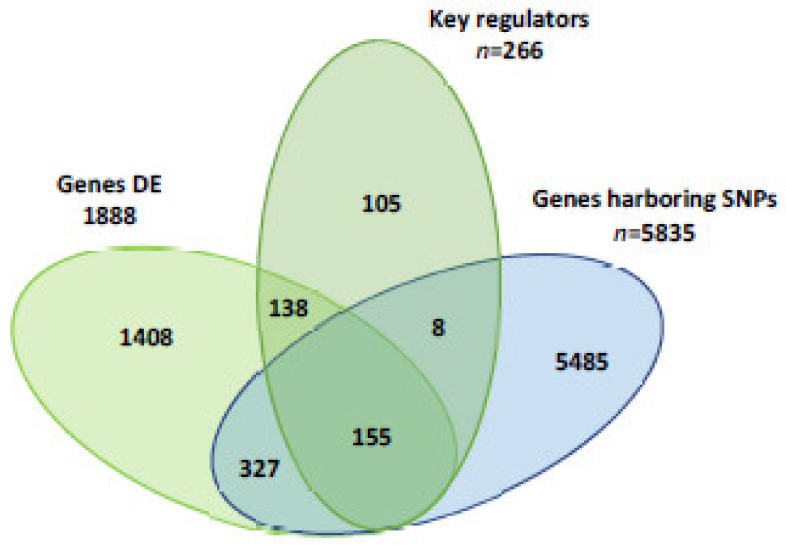
Number of differentially expressed (DE) genes, key gene regulators and genes harboring a single nucleotide polymorphism (SNP) in resistant milk fat depression cows fed the linseed diet compared with sensitive milk fat depression cows fed the linseed diet.

In order to identify those loci involved in the synthesis of FA and lipid metabolism within the context of milk fat depression, the following selective criteria were assumed: (a) differentially expressed genes (RPKM ≥ 0.2, *p*-value < 0.01 and fold change > 2 and <−2); (b) identified as key gene regulators; and (c) containing polymorphic SNP in the two cows R-MFD or S-MFD at the same time. Selected genes containing polymorphic SNP are important because they can be future markers to select cows resistant or sensitive to MFD. These genes in R-MFD cows fed the LIN diet compared with S-MFD cows fed the LIN diet would explain the differences between S-MFD and R-MFD cows, and at the same time the effect of linseed. For these reasons, this comparison would be the most relevant (Figure 1). The selected genes were then used to check the differences among the other comparisons.

The comparison between R-MFD and S-MFD cows provided 1888 differentially expressed genes, 266 key gene regulators and 5835 genes containing polymorphic SNP. Of these, 15 genes met all three criteria (Table 1). Without discarding the influences from other loci, these 15 genes should be viewed as remarkable candidates to be involved in the resistance or sensitivity of dairy cows to MFD. The fold changes for the gene expression of these 15 loci in the other comparisons are presented in Table 2. In general, most genes detected when R-MFD cows were fed the LIN diet compared with S-MFD cows were fed the LIN diet were also detected in R-MFD cows fed the CTR diet compared with S-MFD cows fed the CTR diet. The genes APBB1, EREG, ITGB4, NFATC2, NOTCH1, PROM1, RICTOR, TGFBR3, WWC1 and ZNF217 were down-expressed, and FLT1 was over-expressed in R-MFD cows with the LIN and CTR diet compared with S-MFD cows. These results suggest that R-MFD and S-MFD cows had a similar pattern of gene expression, with or without linseed in the diet. Therefore, it is suggested that R-MFD and S-MFD cows activate different pathways involved in milk fat synthesis always, not only in response to linseed. The gene MTOR was the only gene found down-expressed in R-MFD cows fed the LIN diet, and over-expressed when fed the CTR diet compared with S-MFD. The genes MTOR, NFATC2 and PDPK1 were down-expressed in R-MFD cows compared with S-MFD fed the LIN diet, and in the LIN diet compared with the CTR diet in R-MFD cows. These three genes showed a difference between R-MFD and S-MFD cows, in addition to a response to linseed in R-MFD cows.

**Figure 2:**
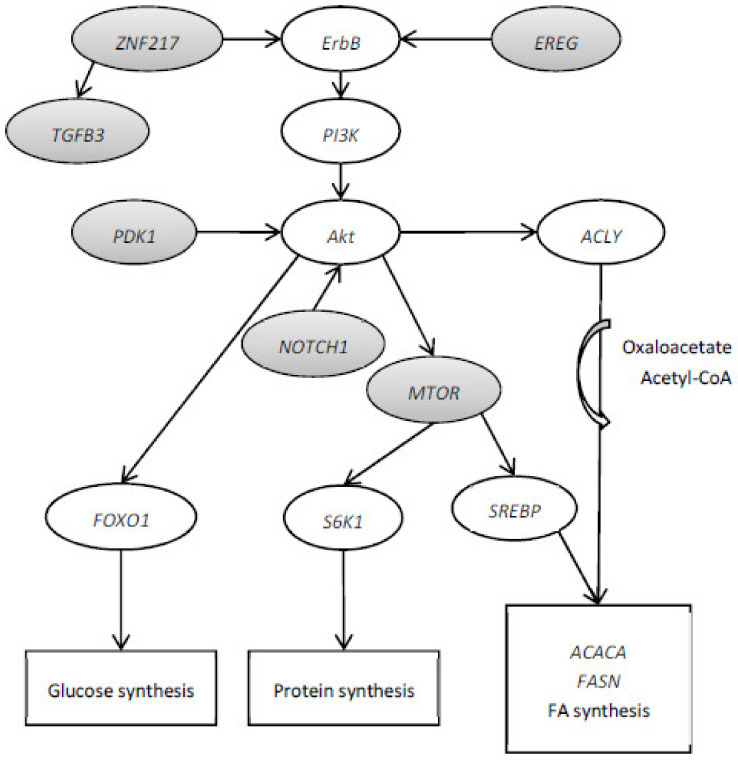
Hypothetical network of novel candidate genes to be involved in the resistance or sensitivity of dairy cows to milk fat depression. Differentially expressed genes, key gene regulators and harboring single nucleotide polymorphism (grey) in resistant milk fat depression cows fed the linseed diet compared with sensitive milk fat depression cows fed the linseed diet.

The mammalian target of rapamycin (MTOR) gene plays a key role in enhancing protein synthesis and cell growth [28]. Moreover, MTOR is also related to lipid biosynthesis by controlling SREBP1 expression [29,30]. Li et al. [31] observed a positive feedback-loop regulation between the SREBP1 and MTOR signaling pathways in dairy cow mammary epithelial cells. The SREBP1 is known to rule milk fat synthesis by activation of the sterol responsive element containing genes involved in de novo FA synthesis such as ACACA and FASN [32]. On the other hand, Portsmann et al. [33] reported that the activation of de novo FA synthesis by the serine/threonine kinase (Akt) signaling pathway requires the MTOR function. The Akt plays a role in glycolysis and FA biosynthesis by activation of ATP-citrate lyase (ACLY) and FASN. On the other hand, ACLY converts cytosolic citrate into acetyl-CoA and oxaloacetate to lipid biosynthesis. The activation of SREBP and Akt-dependent induction of lipid biosynthesis requires the activity of MTOR. Moreover, phosphoinositide-dependent kinase 1 (PDPK1) is related to the phosphorylation and activation of Akt. The gene Akt is suggested to be downstream of MTORC2, which contains mTOR, rapamycin-insensitive companion of mTOR (RICTOR) and GβL [34] (Figure 2).

Portmann et al. [33] observed that the activation of SREBP1 by MTOR requires the activation of PI3K. The entire pathway PI3K/Akt/MTOR may be involved in lipid or protein synthesis. Epiregulin (EREG) is a member of the epidermal growth factor (EGF) family of peptide growth factors. EREG is involved in the PI3K/Akt signaling pathways in HepG2 cells [35,36]. Pajvani et al. [37] observed that hepatic NOTCH1 plays a significant role in glucose (via FOXO1) and lipid (via MTOR) metabolism by its ability to uncouple Akt from MTOR. The insulin-like growth factor 1 (IGF-1) gene has been shown to be associated with milk protein yield, milk fat yield, milk fat concentration, somatic cell score, carcass conformation and carcass fat in Holstein-Friesian dairy cattle [38]. The gene IGF-1 was associated with transcription factors HSF1 and zinc finger protein 217 (ZNF217) [38]. The ErbB3 gene was identified as a direct target for the ZNF217 transcription factor and was the first gene shown to be positively regulated by the recruitment of ZNF217 to its promoter [39]. The effect of ZNF217 on ErbB receptor expression possibly augments PI3K/Akt [39]. The activation of ZNF217 increases the expression of the TGF-β pathway and transforming growth factor beta 3 (TGFB3) promoter [40]. Therefore, the selected genes MTOR, PDPK1, RICTOR, EREG, NOTCH1, ZNF217 and TGFB3 could be possible candidates to form a network with a principal axis PI3K/Akt/MTOR/SREBP1 involved in lipid metabolism (Figure 2).

Dolgacheva et al. [41] observed a relationship between the gene CD38 molecule (CD38) and PI3K/Akt signaling pathway in primary white adipocytes culture when calcium signaling pathways activated by angiotensin II were analyzed.

The gene amyloid beta precursor protein binding family B member 1 (APBB1) was related with an important role in the pathogenesis of Alzheimer’s disease (provided by RefSeq, Mar 2012). Johansson et al. [42] observed that PPAR signaling pathway genes and IGF1 were affected in the microglia in mouse models of Alzheimer’s disease. IGF1 was involved with the PI3K/Akt pathway [42] and PPAR is a transcription factor involved in milk fat synthesis in bovine mammary epithelial [5]. The relationship between PPAR and the PI3K/Akt signaling pathways and Alzheimer’s disease could suggest a slight connection between gene APBB1 and fat synthesis.

Cui et al. [20] analyzed differentially expressed genes between Holstein cows with extremely high and low milk protein and fat percentage. They concluded that VEGF, among others, may be a promising candidate gene involved in controlling milk fat and protein percentage. Rossiter et al. [43] also observed that the gene vascular endothelial growth factor A (VEGF), acts in fully differentiated mammary gland cells in mice. The fms-related tyrosine kinase 1 (FLT1; also known as VEGFR1) is a receptor of VEGF, and it is possible that FLT1 also plays a role in milk fat synthesis. Adini et al. [44] observed a direct relationship between gene prominin-1 (PROM1) and VEGF in primary endothelial and melanoma cells. Ibeagha-Awemu et al. [45] observed that the gene integrin subunit beta 4 (ITGB4) was associated with oleic acid in milk somatic cells of Canadian Holstein cows, and indicated a relationship between ITGB4 and milk traits. No relationship was found among the genes WWC1 and NFATC2 and changes in milk fat synthesis.

## 4. Conclusions

Differential expression analysis detected differentially expressed genes in all the comparisons conducted, except in the LIN vs. the CTR diet in S-MFD cows. These results suggest that R-MFD cows could be activating a compensatory mechanism to increase the FA synthesis in linseed-rich diets. The results identified multiple pathways and key gene regulators differentially expressed between diets and cows. These metabolic pathways and key gene regulators are involved in the immune and inflammatory system, development and growth processes, lipid metabolism and FA synthesis. Finally, 15 genes were detected as differentially expressed genes, key gene regulators and harboring SNP in R-MFD cows fed the LIN diet compared with S-MFD cows fed the LIN diet. These 15 genes are novel candidate genes to be involved in the resistance or sensitivity of dairy cows to milk fat depression. The results suggest that more complicated networks are involved in milk fat synthesis and in the response to diets that induced MFD.

## Figures and Tables

**Table 1:** Genes containing polymorphic single nucleotide polymorphism (SNP) associated with differentially expressed genes and key gene regulators in resistant milk fat depression cows fed the linseed diet compared with sensitive milk fat depression cows fed the linseed diet.

Cow ^1^	Gene	*p*-Value	q-Value	FC ^2^	Chr. ^3^	Chr. Position	SNP ID	Allele Change
S	*APBB1*	0.01	0.04	−3.36	15	45561080	rs207812421	C/T
S	*APBB1*	0.01	0.04	−3.36	15	45561104	rs209491693	T/C
S	*APBB1*	0.01	0.04	−3.36	15	45569813	rs210165374	G/C
S	*APBB1*	0.01	0.04	−3.36	15	45569330	rs210595896	C/T
S	*APBB1*	0.01	0.04	−3.36	15	45569819	rs211146069	T/C
S	*APBB1*	0.01	0.04	−3.36	15	45576807	rs41255144	C/T
S	*CD38*	0.01	0.02	−2.31	6	115589806	rs109641719	C/T
S	*CD38*	0.01	0.02	−2.31	6	115591914	rs134955750	G/A
R	*CD38*	0.01	0.02	−2.31	6	115595101	rs136147162	G/C
S	*CD38*	0.01	0.02	−2.31	6	115592674	rs43434904	C/T
S	*CD38*	0.01	0.02	−2.31	6	115589816	rs43434912	G/A
S	*EREG*	0.01	0.01	−9.50	6	92346668	rs42580620	G/A
R	*FLT1*	0.01	0.01	3.55	12	31554406	rs109247749	A/G
R	*FLT1*	0.01	0.01	3.55	12	31489476	rs111027111	C/T
R	*FLT1*	0.01	0.01	3.55	12	31483700	rs133983660	A/G
R	*FLT1*	0.01	0.01	3.55	12	31485999	rs136560138	G/C
R	*FLT1*	0.01	0.01	3.55	12	31528552	rs137508649	A/G
R	*FLT1*	0.01	0.01	3.55	12	31484135	rs207631114	G/C
R	*FLT1*	0.01	0.01	3.55	12	31483177	rs209090694	T/C
R	*FLT1*	0.01	0.01	3.55	12	31483898	rs209547908	A/G
R	*FLT1*	0.01	0.01	3.55	12	31483715	rs210883339	G/C
R	*FLT1*	0.01	0.01	3.55	12	31483473	rs211512991	T/C
S	*ITGB4*	0.01	0.01	−4.12	19	57146058	rs41926899	G/A
S	*ITGB4*	0.01	0.01	−4.12	19	57157390	rs41927658	C/G
S	*MTOR*	0.01	0.02	−2.18	16	39224720	rs208757293	T/A
S	*MTOR*	0.01	0.02	−2.18	16	39235739	rs211448695	G/A
S	*MTOR*	0.01	0.02	−2.18	16	39231288	rs211677647	C/T
S	*NFATC2*	0.01	0.01	−6.51	13	80208242	rs137043317	T/C
S	*NOTCH1*	0.01	0.02	−2.47	11	107708189	rs110163085	C/G
R	*NOTCH1*	0.01	0.02	−2.47	11	107686385	rs133307736	A/G
S	*NOTCH1*	0.01	0.02	−2.47	11	107685263	rs207760072	A/G
S	*NOTCH1*	0.01	0.02	−2.47	11	107698629	rs211580903	G/A
S	*NOTCH1*	0.01	0.02	−2.47	11	107679366	rs378232535	C/T
R	*NOTCH2*	0.01	0.03	−2.51	3	25061073	rs135438495	T/C
S	*PDPK1*	0.01	0.02	−3.58	25	2633350	rs208965123	T/C
S	*PROM1*	0.01	0.01	−17.9	6	115755844	rs110069470	A/G
S	*PROM1*	0.01	0.01	−17.9	6	115732932	rs42165955	A/C
S	*RICTOR*	0.01	0.01	−2.90	20	37609145	rs41940570	T/C
S	*RICTOR*	0.01	0.01	−2.90	20	37610024	rs41940571	A/G
R	*TGFBR3*	0.01	0.01	−3.90	3	54795884	rs110491344	A/G
R	*TGFBR3*	0.01	0.01	−3.90	3	54796526	rs134330950	T/C
R	*TGFBR3*	0.01	0.01	−3.90	3	54798328	rs379514543	A/C
R	*WWC1*	0.01	0.01	−7.19	7	82262781	rs108980081	C/T
S	*ZNF217*	0.01	0.05	−2.06	13	82061022	rs134599263	A/G

^1^ Cow: S = sensitive to milk fat depression cows; R = resistant to milk fat depression cows. ^2^ Fold change. ^3^ Chromosome.

**Table 2:** Fold Change (FC) of selected genes containing polymorphic single nucleotide polymorphism in cows resistant to milk fat depression and fed the linseed diet compared with cows sensitive to milk fat depression and fed the linseed diet among the other comparisons.

	Comparisons (FC) ^1^			
Gene	1	2	3	4
*APBB1*	−3.36	−6.42	- ^2^	-
*CD38*	−2.31	-	-	-
*EREG*	−9.50	−3.13	-	-
*FLT1*	3.55	5.95	-	-
*ITGB4*	−4.12	−4.97	-	-
*MTOR*	−2.17	RG ^3^ 1.025	-	GDE ^4^ −2.40
*NFATC2*	−6.51	RG −2.17	-	GDE −3.58
*NOTCH1*	−2.47	−3.52	-	-
*NOTCH2*	−2.51	-	-	-
*PDPK1*	−3.58	-	-	GDE −3.88
*PROM1*	−17.89	GDE −12.72	-	-
*RICTOR*	−2.90	GDE −2.47	-	-
*TGFBR3*	−3.90	GDE −2.36	-	-
*WWC1*	−7.19	GDE −6.74	-	-
*ZNF217*	−2.06	−2.77	-	-

^1^ (1) Resistant milk fat depression cows fed the linseed diet compared with sensitive milk fat depression cows fed the linseed diet; (2) resistant milk fat depression cows fed the control diet compared with sensitive milk fat depression cows fed the control diet; (3) sensitive milk fat depression cows fed the linseed diet compared with sensitive milk fat depression cows fed the control diet; and (4) resistant milk fat depression cows fed the linseed diet compared with resistant milk fat depression cows fed the control diet. ^2^ Gene not detected as a differentially expressed gene neither as a key gene regulator. ^3^ Gene detected only as a key gene regulator. ^4^ Gene detected only as a differentially expressed gene.

## Data Availability

Data presented in this study are available upon request from the corresponding author. The data are not publicly available due to a current study for other purposes in a confidentiality agreement.
